# Symbiosis takes a front and center role in biology

**DOI:** 10.1371/journal.pbio.3002571

**Published:** 2024-04-05

**Authors:** Margaret McFall-Ngai

**Affiliations:** Biosphere Sciences and Engineering, Carnegie Institution for Science, and Biology and Biological Engineering, California Institute of Technology, Pasadena, California, United States of America; University of Texas Austin, UNITED STATES

## Abstract

Research enabled by recent technological advances has revealed that all animals and plants likely require interactions with microbes, often in strong, persistent symbiotic associations. This Perspective article argues that while the recognition of this phenomenon is slow in coming, it will impact most, if not all, subdisciplines of biology.

## Teaser

All animals and plants likely require interactions with microbes, often in strong, persistent symbiotic associations. While the recognition of this phenomenon has been slow in coming, it will impact most, if not all, subdisciplines of biology.

Three major revolutions have occurred in biology over the last 150+ years: in the 19th century, the development of the theory of evolution by natural selection; in the 20th century, the discovery of DNA as the genetic material of all life forms; and in the 21st century, the recognition of the primacy of the microbial world. This most recent revolution was a result of the integration of the first 2. It began in the 1970s with Carl Woese’s work [1], in which he used nucleic acid sequences to determine the evolutionary relationships between microbes; however, at that time, the technology was slow and expensive. Advances in nucleic acid chemistry around 2006 (i.e., “next-generation sequencing”), rendered these determinations rapid and inexpensive, thereby democratizing the process. This breakthrough catalyzed the development of a new perspective on microbiology, revealing a world we could not have known. Efforts such as the NIH Human Microbiome Project (2007 to 2016), the Tara Oceans Project (2009 to 2013), and the Earth Microbiome Project (2010 to present) have demonstrated that the vast majority of the biosphere’s diversity has been, over evolutionary time and up to the present day, strongly dominated by microbes. Those life forms that we can see with the naked eye occur as a patina on this unseen tapestry of vast physiological and ecological range and impact.

One major change that has resulted from this revolution is the recognition of the widespread occurrence of animal and plant symbioses with microorganisms. The scholarly books of AE Douglas, *Symbiotic Interactions* (1994) and *The Symbiotic Habit* (2010), which were written before and after the onset of the “revolution,” respectively, illustrate this point. It had been thought that maintaining a lifelong relationship with a specific set of microbes was largely restricted to invertebrate and plant species. The emerging capability to identify microbial species has demonstrated that symbiosis among these taxa is even more pervasive than had been appreciated; however, the most striking change in our understanding has been with the vertebrates. The current data show that acquisition, development, and maintenance of a taxon-specific microbial consortium along mucosal surfaces of most vertebrate organ systems is a shared, derived character of all jawed vertebrates, not only of ungulates. These symbiotic communities interact both directly with host tissues and indirectly through the import of metabolic products of the microbiota into the host’s metabolome (i.e., the small molecules in the blood and tissues) [2]. Through these influences, these microbial consortia profoundly influence the form and function of the healthy individual host animal and, when dysfunctional, are often the root cause of disease.

Since enabled by technology in ~2006, the incorporation into biological thought of this new view of all animals and plants as deeply nested within, and reliant upon, the microbial world has been underway, as evidenced by an exponential increase in publications. Seventy-five percent of the references in NCBI/PubMed to the search word “symbiosis” (first present in 1913) have been published since 2006, as have 99% of the references to “microbiota” (first appearing in 1956). This new knowledge base will change subdisciplines of biology to varying degrees. Some areas, such as biomechanics, are likely to be little affected, but others, including immunology and developmental biology, evolutionary biology, and neurobiology, are already experiencing a reshaping as symbiosis is incorporated into their research frameworks.

Consider, for example, our concepts of the animal immune system. While invertebrates have only the components of an innate immune system, the possession of both an innate and an adaptive immune system is a shared, derived character of all vertebrate species. Immunologists had long believed the principal selection pressure on both these systems was to keep the onslaught of pathogens at bay. Specifically, immunity operated as a strong mechanism, often with a memory, for reacting to adverse interactions with the microbial world; the many microbes that were believed to have no effect on the host (i.e., “commensals”) were simply ignored. With an increase in research on the human microbiome, the view of the vertebrate immune system has reversed [3]; it is now perceived as a mechanism that manages the large, complex “ecosystem” of beneficial microbes that, while composed of human-associated guilds, are unique, like fingerprints, to each individual [4]. In fact, the realization that a person’s microbiome provides a recognizable signature is driving its application in forensic sciences [5]. Thus, to operate effectively, the vertebrate immune system must be permissive and flexible, maintaining a highly complex set of microbes in balance while detecting and attacking pathogens. Vertebrates arose early in animal evolution and only represent 3% of its diversity; therefore, the coordination of an innate immune system with an adaptive immune system is not only a unique strategy, but also a rather rare one among successful animal species.

The immune system of invertebrates, which comprise the other 97% of animal species, is not as well understood as that of vertebrates. All metazoans, from anemones and flatworms to insects, urchins and clams, share with the vertebrates an innate immune system that exhibits many biochemical, molecular, and physiological similarities (i.e., innate immunity is conserved across the evolution of animals). However, innate immunity had also been assumed to have no memory [6] and to be nonspecific:

*“The innate immune system is the body’s first line of defense against germs entering the body*. *It responds in the same way to all germs and foreign substances*, *which is why it is sometimes referred to as the ‘nonspecific’ immune system*.” NIH Website, 2020

New data are calling these assumptions into question for vertebrates [6] as well as invertebrates; for example, in both these animal groups, elements of the innate immune system (e.g., antimicrobial peptides, microbe-associated molecular patterns) participate in shaping the symbiotic microbiota [7]. However, studies of the mutualistic symbioses between microbes and invertebrates have demonstrated that the actions of the innate immune system are very different from those in vertebrates. While often living in environments with dense microbial assemblages, such as the soil or oceans, many invertebrates can recruit and maintain stable and invariant associations with a very limited number of microbes. For example, in the squid–vibrio system on which my lab works, the male host harbors a single, specific, vibrio species [8]. Within a few hours after hatching from the egg into the surrounding seawater, which contains about a million nonspecific bacteria per milliliter, the juvenile squid harvests its specific microbial symbiont, which represents <0.1% of the bacterioplankton. The host squid retains its dynamic vibrio populations over its lifetime, and confocal analyses of living animals has demonstrated that all other tissues, both within the mantle cavity and on the surfaces, remain free of living microbes of other phylotypes. This behavior of specific host–symbiont recognition occurs with fidelity each generation, approximately 4 generations a year, and has done so for tens of millions of years. We know that the innate immune system controls some of this process. How much is controlled by other, unknown activities that involve a type of recognition memory remains to be determined. However, the processes that control colonization and persistence in this association cannot possibly be viewed as nonspecific.

More recently, emerging studies (e.g., [9,10]) of several other associations have discovered similar patterns of a limited, predictable set of stable symbiont species living with an invertebrate host. However, it should be noted that exceptions to this general strategy have been documented. For example, termites, cockroaches, and sponges have highly diverse microbiota that can consist of hundreds of species. At the other end of the spectrum, it is likely that some animals do not harbor a persistent microbiome [11]; nevertheless, they still may have essential interactions with specific microbes at certain periods in their ontogeny (e.g., [12]). Finally, controversy still exists surrounding the actual nature of certain symbioses, such as those of corals [13,14], concerning whether or not the microbiomes of these invertebrate species are highly diverse.

These are early days and we have barely scratched the surface of the vast diversity of symbiotic systems that drive the biosphere. The ecological niches filled by invertebrates and plants are so varied that many strategies for living in the microbial world remain to be discovered. To address these different systems with the highest possible rigor, strong collaborations between animal and plant biologists and the community of microbiologists will be essential, although this imperative will not be easy, as the fields have been in silos since the 19th century. Despite these cultural challenges, it is a new day for biology, with a vast frontier to explore.
